# A randomised investigation of journal responses to academic and journalist enquiry about possible scientific misconduct

**DOI:** 10.1186/s13104-018-3613-1

**Published:** 2018-07-30

**Authors:** Mark J. Bolland, Alison Avenell, Greg D. Gamble, Stephen Buranyi, Andrew Grey

**Affiliations:** 1 Bone and Joint Research Group, Department of Medicine, Faculty of Medical and Health Sciences, University of Auckland, Private Bag 92019, Auckland, 1142 New Zealand; 2 Health Services Research Unit, University of Aberdeen, Foresterhill, Aberdeen, Scotland AB25 2ZD UK; 3 London, UK

**Keywords:** Misconduct, Retraction, Research fraud

## Abstract

**Objective:**

We investigated whether responses about possible scientific misconduct from journals to journalists would differ in speed, usefulness, and tone from responses to academics. Twelve journals that published 23 clinical trials about which concerns had been previously raised were randomly assigned to enquiries by a journalist or academics. Emails were sent every 3 weeks to the journal editor. We recorded the time for the journal to respond, and two investigators independently assessed the usefulness and tone of the journal responses.

**Results:**

10/12 journals responded: 3 after one email, 5 after two emails, and 2 after three emails (median time from first email to response: 21 days; no difference in response times to journalist or academics, P = 0.25). Of the 10 responses, 8 indicated the journal was investigating, 5 had a positive tone, 4 a neutral tone, and 1 a negative tone. Five of the enquiries by the academics produced information of limited use and 1 no useful information, whereas none of the 6 journalist enquiries produced useful information (P = 0.015). None of the 10 responses was considered very useful. In conclusion, journal responses to a journalist were less useful than those to academics in understanding the status or outcomes of journal investigations.

**Electronic supplementary material:**

The online version of this article (10.1186/s13104-018-3613-1) contains supplementary material, which is available to authorized users.

## Introduction

When scientific misconduct occurs, the relevant literature ought to be promptly corrected. However, there are often lengthy delays between concerns being raised and formal actions such as expression of concern notices or article retraction (see Table 1 for description). For example, concerns were raised about the work of the Japanese anaesthetist Yoshitaka Fujii in 2000 [1] but it took until 2012 before the body of work was publicly confirmed as fraudulent leading to the recommendation that 183 randomised controlled trials (RCTs) be retracted [2, 3]. Delays may occur for many reasons, but one common theme is that formal investigations take a very long time. The average duration of investigation by the Office of Research Integrity between 2001 and 2010 was 20 months, with some investigations lasting at least 9 years [4]. Other concerns raised about the management of scientific misconduct include uninformative retraction notices and failure to retract articles even when there is clear evidence of misconduct [4].

**Table 1:** Definition of scientific misconduct and COPE guidelines for journal responses to errors or misconduct (Adapted from [6])

Definition of scientific misconduct	
Fabrication, falsification, or plagiarism in proposing, performing or reviewing research, or in reporting research results [5]	

COPE guidelines	
Problem	Recommended action
A small part of otherwise reliable publication is misleading (especially if honest error)	*Correction*
Incorrect author list	
Inconclusive evidence of research or publication misconduct	*Expression of concern*
Evidence that findings are unreliable but authors’ institution will not investigate	
Believe an investigation has not been, or would not be, fair and impartial or conclusive	
An investigation is underway but results not be available for some time	
Clear evidence that findings are unreliable because of misconduct or honest error	*Retract publication*
Findings have been published previously	
Plagiarism	
Research is unethical	

*COPE* Committee on Publication Ethics

Beginning in March 2013, we started to report to affected journals numerous concerns about a set of 33 RCTs from a group in Japan, including objective statistical evidence of implausible characteristics of randomised treatment groups, improbable recruitment rates and implausibly positive outcome data, lack of ethical oversight, plagiarism and many logical and other errors. Between October 2015 and September 2016, 10 RCTs were retracted based upon these concerns. The reasons for retraction included scientific misconduct, concerns about data integrity, fraud, extensive self-plagiarism and honorary authorship. In November 2016, our systematic review describing the concerns about the RCTs was published [7]. An accompanying editorial stated that the lead author admitted that the three RCTs in that journal were fraudulent, and that the editors of the journals that published the remaining RCTs had been notified of the concerns [8]—these notifications occurred in September 2016.

We expected that a number of other retractions would follow this publication and the journal notifications, but 4 months later we had received no new information and nothing further had happened in public. The journal that published our systematic review indicated that it had completed its involvement. Therefore, we planned to contact each journal with unretracted RCTs to ask for an update, in the hope of expediting processes to preserve the integrity of the research literature. Previously, we had found that our enquiries to journals about their investigations of our concerns generated responses of variable timing, usefulness and tone. We wondered if it would make any difference whether the enquiry to the journal came from an academic group or a journalist. Therefore, we invited a journalist (SB) who has recently investigated and written about scientific misconduct and the world of academic publishing for the *Guardian* newspaper [9–11] to take part in a randomised comparison of journal responses to contact by academics or a journalist. Specifically, we hypothesized that journal responses to journalists would differ in speed, usefulness, and tone from journal responses to academics.

## Main text

### Methods

We contacted the editors of the 12 journals that published the 23 unretracted trial publications using email contact details on the journal website, or where these were not available or not responded to, using email details obtained from an internet search. Each journal was randomised to receive a standard letter from the journalist or from our group of academics (Additional file 1: Appendix S1). Journals were randomised in two blocks, one block for each of the two first authors on the 23 publications, using random numbers generated with Excel 2010. Thus, six journals with between 1 and 3 publications each (total 12 publications) were sent a letter from the journalist and six journals with between 1 and 4 publications each (total 11 publications) were sent a letter from the academics. Each journal editor was contacted contemporaneously by email, and if no response was received within 3 weeks, a follow-up email was sent. We sent a maximum of 3 emails in total.

We recorded basic facts about each of the journals, including the publisher, impact factor, and whether the journal was a member of the Committee on Publication Ethics (COPE), who provide guidance for dealing with scientific misconduct, or had previous experience with retractions, as determined by Pubmed and Google searches. For each journal, we recorded the time taken to respond and any details provided about investigations being undertaken. Two authors blinded to randomisation (AG, AA) independently classified the usefulness of information provided in the responses, and the tone of the journal response. We pre-specified that a very useful response would clearly state what the journal had done to date and the current status of its investigation; whereas a response of no use would not indicate what the journal had done nor the current status of the investigation, and a response of limited use would lie between these two categories. Tone of the response, defined by the Oxford English dictionary as “The general character or attitude of a piece of writing”, was classified as positive, negative or neutral by each investigator according to their own judgement. Agreement of these classifications between authors was 75%-kappa statistic for usefulness 0.64, and for tone 0.53. In cases of disagreement, the independent assessment of a third author (MB) acted as a tiebreaker, with final categorisation agreed by consensus.

We compared the median time to a response using the log-rank test and the differences in usefulness and tone with Fisher’s Exact test (GraphPad Prism version 7.03 for Windows, GraphPad Software, La Jolla California USA, https://www.graphpad.com). P < 0.05 was considered statistically significant.

Due to the nature of the study, ethical approval was not considered necessary.

### Results

The 23 unretracted trial publications were published in 12 journals from 8 different publishers (Additional file 1: Appendix Table S1). Three journals are open access, 7 are members of COPE, 11 have an impact factor, which ranges from 1.2 to 5.79, and 8 have prior experience with retractions.

Ten of the 12 journals responded, 3 after the first email, 5 after the second email, and 2 after the third email. 8 journals responded within 1 day of the most recent email being sent. Thus, the median time from the first email to a response was 21 days. There was no difference in response times between the two groups (P = 0.25).

Table 2 shows details of the responses received (Additional file 2). Of the 10 responses, 8 indicated that the journal was investigating, 4 whether or not the lead author had been contacted, and 1 whether or not the institution had been contacted. Only 3 responses indicated that the journal would be in contact in the future, but none made contact within 5 months of the journal’s response.

**Table 2:** Journal responses

	Journal contacted by Academics (n = 6)	Journal contacted by Journalist (n = 6)
Publications (n)	11	12
Response from journal	6	4
Response indicates: (Yes/no/not stated)		
Journal is investigating	6/0/2	2/0/2
Author contacted	3/1/2	0/0/4
Institution contacted	0/1/5	0/0/4
Journal will be in contact in future	1/2/3	2/0/2
Consensus assessment		
Tone (positive/neutral/negative)	4/2/0	1/2/1
Information obtained (useful/ limited use/no use)	0/5/1	0/0/6^a^

^a^Includes 2 journals who did not respond to 3 emails

Overall, we considered that 5 of the 6 enquires made by academics produced information of limited use and 1 no useful information, whereas none of the 6 enquiries by the journalist produced useful information (P = 0.015). None of the 10 responses were considered to be very useful. Table 3 has examples of the responses and their classification. The tone of the journal reply was positive for 5 responses, neutral for 4 responses, and negative in 1 response. There was no difference between the proportion of positive/neutral (versus negative) responses to the academics and the journalist (P = 0.40).

**Table 3:** Examples of journal responses and classification

Response	Classification
We have no comment	Negative tone/no use
Your email was forwarded to me. We’re still looking into this matter per our policies	Neutral tone/no use
I have forwarded your e-mail to the editor and will keep you posted regarding the same	Neutral tone/no use
Sorry for the delay in responding—for some reason your earlier emails did not get through to me… we have been working on this issue for some time now. The … managing editor … has been working with … staff as there is a defined process they need to follow. She is looking into where we stand with this and I will follow up with you once I learn more	Positive tone/no use
Thanks very much for your message regarding the two papers published in … Yes, the … has initiated an investigation using the COPE guidelines. The results of this investigation will guide our future actions	Positive tone/limited use

One of the 23 publications was retracted because of scientific misconduct in the 5 months after our initial emails (Additional file 1: Appendix Table S1), but no public statements or expressions of concern were issued about any of the remaining 22 publications during this period.

### Discussion

Journals responded to enquiries by academics with more useful information, (although that information was still of limited use), than they provided to the journalist, but there were no differences in the tone or speed of the journal response to the academics or journalist. These findings were contrary to some of our expectations. Prior to the study, we had a range of views as to whether there would be differences in journal responses to being contacted by a journalist or by a group of academics, in general expecting either no differences or that the journalist would receive faster responses.

Only 25% of journals (3/12) responded to the initial email contact, and 17% (2/12) did not respond despite being sent 3 separate emails. When journals did reply, the response was quick: 8/10 responses came within 1 day of the most recent email, and the other two within 8 days of the most recent email. However, the information provided by the journals was of limited or no use in understanding what was happening. While 8/10 responses indicated that an investigation was taking place, only 4 indicated whether or not the author had been contacted and only 1 whether or not the institution had been contacted. Three responses stated that we could expect further contact from the journal, but none gave an indication of the expected time frame and no journal has contacted us as yet. Despite our enquiries, the investigations of the possible misconduct did not appear to have proceeded: in the 11 months after the journals were all first notified by another journal editor about the possible scientific misconduct (including the 5 months after our first email) only one journal made any public statement about the integrity of an RCT—it was retracted.

Unbeknown to us, a journalist from the Retraction Watch website also contacted the journals with the unretracted papers between our 2nd and 3rd email contacts [12]. Retraction Watch is a prominent website that publicly records and comments upon scientific misconduct, and regularly corresponds with journals about misconduct. Therefore, its journalists might be expected to obtain more useful information more frequently than other journalists or academics. Of the 12 journals potentially contacted, 7 responded to Retraction Watch. Applying the same classifications we used, 2 responses were very useful, 2 of limited use, and 3 of no use. One response stated that the journal, a member of COPE, did not investigate issues of misconduct. This independent attempt at contacting journals confirms that journals often do not respond, and when they do, the information provided is usually of limited or no use.

Our study has highlighted an important problem. When there is clear-cut evidence of research misconduct identified in previous investigations, there seems little reason for long delays or a reluctance to provide useful information about the processes being undertaken to correct the scientific record, nor to promptly publish an expression of concern. But even after recommendation for retraction following an official investigation by a German State Medical Association, 10% of articles remained unretracted after 2 years [13]. Failure to express concern or retract articles means that patients and research participants may be put at risk if they receive treatment based on findings that are later retracted because they were incorrect or unreliable and research funds may be wasted by exploring hypotheses based on invalid data.

## Limitations

The major limitations to our findings arise from the necessarily small and selected group of journals contacted. By necessity, the study focused only on a group of 23 RCTs published in 12 journals. These RCTs were part of a broader group of 33 RCTs about which concerns regarding possible scientific misconduct had been published [7]. All the affected journal editors had been notified of the concerns by the editor of the journal that published these concerns [8]. It would be valuable to repeat the study with a larger number and broader range of journals. However, potential widespread scientific misconduct is rare, and an opportunity for independent investigators to repeat our study might not occur for some time.

## Additional files

**Additional file 1.** Template for letters from academics to journalist to journal editors. **Table S1.** References of 23 randomised controlled trials.

**Additional file 2.** Study data. Contains all study data.

## Abbreviations

RCT: randomised controlled trial
COPE: Committee on Publication Ethics
