# Supplementary material for: A randomised investigation of journal responses to academic and journalist enquiry about possible scientific misconduct
Source: BMC Res Notes. 2018 Jul 30;11:521. doi: 10.1186/s13104-018-3613-1 (PMC6065063; doi:10.1186/s13104-018-3613-1)
Supplement: Supplementary file 2 — Additional file 2. Study data. Contains all study data. [file 13104_2018_3613_MOESM2_ESM.pdf]

			Journal characteristics						
**Journal**	**Number of publications**		**Impact Factor**	**Publisher**	**Country of publication (Special society/ publisher)**	**Open Access (Y/N)**	**COPE Member**	**Previous experience with retractions**	
Stroke	1		5.79	Lippincott williams and wilkins	USA	N	Y	Y	
J Stroke Cerebrovasc Dis	2		1.6	Elsevier	USA/Japan	N	Y	N	
J Neurol Neurosurg Psychiatry	3		6.4	BMJ	UK	N	Y	Y	
J Neurol Sci	2		2.1	Elsevier	Netherlands	N	Y	Y	
Cerebrovasc Dis	1		3.4	Karger	Switzerland	N	N	Y	
Bone	4		3.7	Elsevier	Multinational	N	Y	Y	
Kurume Med J	1		Not available	Kurume University School of Medicine	Japan	Y	N	N	
Am J Phys Med Rehabil	2		2.1	Lippincott williams and wilkins	USA	N	Y	N	
Aging Clin Exp Res	1		1.4	Springer	Multinational	N	N	Y	
Yonsei Med J	3		1.2	YONSEI UNIV COLL MEDICINE	Japan	Y	N	Y	
J Musculoskelet Neuronal Interact	1		1.6	JMNI	Greece	Y	N	N	
Osteoporos Int	2		3.4	Springer	Multinational	N	Y	Y	

Journal response
**Response 1**
Your email was forwarded to me. We're still looking into this matter per our policies (see link below).<br>http://www.ahajournals.org/site/misc/EthicalConductPolicy.pdf
I hope this email finds you well. Yes. I have relayed your message to our Editor. They are looking into this. Please be advised we will be in touch as soon as he responds.
We have no comment.
We have received your e-mail - thank you very much! We discussed this issue already in our Editorial Office and contacted the first author of the mentioned paper.
Sorry for the delay in responding – for some reason your earlier emails did not get through to me – perhaps they ran afoul of the [redacted] filter.<br>Regardless, we have been working on this issue for some time now. The BONE managing editor, [redacted], has been working with Elsevier staff as there is a defined process they need to follow. She is looking into where we stand with this and I will follow up with you once I learn more.
Thank you for your e-mail and raising your concern regarding the paper by [redacted] published in the Kurume Medical Journal. We received an inquiry from The Neurology® editorial office in the fall of 2016, expressing concerns about the validity of paper written by [redacted]. Members of the Editorial Board met several times to discuss the issue, and the paper has been retracted as of December, 2016. Technical process of the retraction is underway both on printed and online versions of the Kurume Medical Journal. The Retraction notice will appear on Vol.63, 1&2 later this month. Thank you again for expressing concerns about [redacted] paper and notifying us.
Thanks very much for your message regarding the two papers published in the AJPM&R by [redacted]. Yes, the AJPM&R has initiated an investigation using the COPE guidelines. The results of this investigation will guide our future actions.
I have forwarded your e-mail to the editor and will keep you posted regarding the same.
Thank you very much for your email expressing your concerns about the paper published in the Journal of Musculoskeletal and Neuronal Interactions entitled [redacted]. I have carefully read both the above article and yours in Neurology. For that reasons, I asked the statisticians of my team to read through the published article to JMNI. Unfortunately, from the existing published data on the article (we do not have their primary data), we are unable to have a concrete conclusion about the integrity of this paper or to have indications and evidence that the authors fabricated and falsified the data.<br>However, if you have specific facts about the integrity of the aforesaid article, I kindly ask you to inform me.
Thank you for your letter concerning the papers where[redacted] appears in the authorship of papers published in Osteoporosis International. After extensive review with the senior author and [redacted] we have no evidence of fraud, and indeed evidence to the contrary. By the unanimous decision of the editors we have informed the authors that our investigation is now closed.

**Our reply**
Thanks so much for your quick response and for letting us know what is happening. We appreciate that such investigations are time-consuming, difficult and complicated. Do you have any idea (even a rough guess) of when your investigation might be complete?
Thanks very much for your reply. We have not received any information from any of the journals/institutions that have conducted investigations into the work of [redacted]. So we can only look at the papers, particularly in the context of the authors’ other work, some of which has been publicly confirmed as fabricated.<br>We have conducted a detailed review of this trial, which we think raises a number of important concerns that were also a feature of the authors’ other published trials. The attached document outlines these concerns, which include concerns about author contributions, inclusion and exclusion criteria, randomisation, ethical oversight, improbable baseline data for both anthropometric measurements and laboratory measurements, and implausibly positive outcomes. Collectively, we believe these concerns, particularly in the context of the authors’ body of published work, raise serious doubts about the integrity of the paper that should be investigated. I have also attached the systematic review of whole body vibration referenced in our review with the relevant sections highlighted.<br>[redacted] We hope this will be of use in your investigation of the integrity of this paper.

**Response 2**		
Thanks for your message. I have tried to communicate with [redacted] without success. [redacted].I hope we can complete this in the next couple of months.		
Firstly, I would like to thank you for your descriptive and informative email.<br>I am going to investigate this issue but due to some internal administrative changes, maybe it will take some extra time.<br>I really appreciate your assistance.		

Details												
Response from journal (Y/N)	After how many emails	Time to response after email (days)	Time to total response (days)	Investigation initiated (Y/N/not stated)	Author(s) contacted (Y/N/not stated)	Institution contacted (Y/N/not stated)	Journal will contact at later date (Y/N)	Further contact from journal (Y/N)	Time to Expression of Concern	Reason(s) for EoC	Time to retraction	Reason(s) for retraction
Y	2	0	21	Y	NS	NS	NS					
N												
Y	2	1	22	Y	NS	NS	Y	N				
Y	2	1	22	NS	NS	NS	NS					
Y	2	0	21	Y	Y	NS	NS					
Y	3	1	43	Y	NS	NS	Y	N				
Y	2	0	21	Y	NS	NS	N				23	Scientific misconduct
Y	1	1	1	Y	Y	NS	NS					
Y	1	1	1	NS	NS	NS	Y	N				
N												
Y	3	7	49	Y	N	N	NS					
Y	1	8	8	Y	Y	NS	N					

	Consensus					
	**Tone of response**	**Final information obtained from response (s)**		**Randomised group**		
	Neutral	No use		Journalist		
		No use		Journalist		
	Positive	No use		Journalist		
	Negative	No use		Journalist		
	Positive	Limited use		Academic		
	Positive	No use		Academic		
	Positive	Limited use		Academic		
	Positive	Limited use		Academic		
	Neutral	No use		Journalist		
		No use		Journalist		
	Neutral	Limited use		Academic		
	Neutral	Limited use		Academic		

**Retraction Watch comment**	**Classification**
The American Heart Association journals do not investigate issues of misconduct. Questions such as these should be directed to the researcher's institution.	No use
I forwarded this to Elsevier administration when you first alerted us (JNS). It is in their hands at this time but I do expect some action.	No use
On behalf of the Editor and the Publisher, I wanted to drop you a quick note to let you know that at the moment I don't have any comment on the inquiry below but I will be sure to alert you if we decide to take any action on these publications.	No use
We received an inquiry from The Neurology® editorial office in the fall of 2016, expressing concerns about the validity of paper written by [redacted]. Members of the Editorial Board met several times to discuss the issue, and the paper has been retracted as of December, 2016. Technical process of the retraction is underway both on printed and online versions of the Kurume Medical Journal. The Retraction notice just appeared on Vol.63, 1&2, the most recently published journal. https://www.jstage.jst.go.jp/article/kurumemedj/57/4/57_4_117/_article	Limited
I have personally checked the article published in Aging Clinical and Experimental Research and had an intensive correspondence with [redacted], Executive Editor, *Neurology* Journals, back in September 2016. [redacted] guaranteed to me that after a final check of the paper, whose lead author was [redacted], would have not ended up in the final appendix, because all information collected indicated that the work was correctly done.	Limited
In regards to the suspected fabrication of data found in the following articles, the YMJ editorial committee requested a corresponding author's reply at the end of September last year. So, we received the reply from the corresponding author ([redacted]). After reviewing [redacted] explanation, we found that his reply was clear, therefore, we decided not to take any actions regarding the statuses of these manuscripts at that time. However, our editorial committee will continue to monitor this issue.	Very useful
We have looked into this issue and can confirm that the editors of the journal *Osteoproosis International* have extensively reviewed the papers where [redacted] appears in the authorship. No evidence of fraud was found as a result of the investigation and the editors now consider this case closed.	Very useful
