# Supplementary material for: A randomised investigation of journal responses to academic and journalist enquiry about possible scientific misconduct
Source: BMC Res Notes. 2018 Jul 30;11:521. doi: 10.1186/s13104-018-3613-1 (PMC6065063; doi:10.1186/s13104-018-3613-1)
Supplement: Supplementary file 1 — Additional file 1. Template for letters from academics to journalist to journal editors. Table S1. References of 23 randomised controlled trials. [file 13104_2018_3613_MOESM1_ESM.docx]

**Appendix S1: Letter template for journalist letters and academic author letters**

|   Bone and Joint Research Group  Clinical Research Centre  Level 5, Building 502  Faculty of Medicine and Health Sciences  Private Bag 92019  Auckland 1142  New Zealand  Telephone ----------------  Facsimile: ----------------  Email: ____________ |
| --- |
|  |
|  |
|  |
|  |
|  |
|  |

Academic author letter

Dear Professor

Editor in Chief

[journal name]

[date]

Re: [insert citation(s)]

We write to express concern about the integrity of the above paper(s), published in [name of journal] (attached).

These concerns are based on our systematic review of 33 randomized trials reported by [redacted]. This review was published recently in *Neurology* (attached). In short, the body of work by [redacted] is characterized by strong statistical evidence of failure of randomization, remarkably high productivity and rapid participant recruitment rates, outcome data inconsistent with those of other investigators, textual inconsistencies and replication, data errors, and concerns about ethical oversight.

To date, 10 of the trial reports have been retracted, after the journal involved reviewed the evidence contained in our review and initiated investigations. During these processes, [redacted] acknowledged misconduct in some of the retracted papers, and it was stated that some papers involved ‘honorary authorship’ for co-authors, with one retraction notice stating that the co-authors “contributed little to the published study in this journal” (Parkinsonism Relat Disord. 2016;28:171.)

We think the integrity of the data and authorship in the above named paper(s) published in [name of journal], which was/were included in our review, should be investigated. We understand that the editorial staff of *Neurology* communicated with you about this issue. We would be grateful if you were able to provide an update on the status of these publication(s) and any investigations that [journal] is undertaking.

We look forward to your reply.

Yours sincerely

Associate Professor Mark Bolland

Associate Professor Andrew Grey

Mr Greg Gamble

Professor Alison Avenell

[contact details]

Journalist letter

Professor

Editor in Chief

[journal name]

[date]

My name is Stephen Buranyi. I’m a journalist interested in the use of statistical methods to detect possible research misconduct. I recently wrote an article about this in the Guardian newspaper in the United Kingdom (<https://www.theguardian.com/science/2017/feb/01/high-tech-war-on-science>). I’m currently researching a follow-up story on this topic.

During my research, I came across the attached review in Neurology (attached) on the work of [redacted]. Currently, 10 of the trial reports mentioned in that article have been retracted. [Journal name] published [xx] papers [insert citation(s)] that were included in the Neurology review. The associated editorial (attached) mentions that the editor of Neurology contacted you about this issue.

I would be grateful if you were able to provide an update on the status of these publication(s) and any investigations that [journal] is undertaking.

Kind regards

Stephen Buranyi

[contact details]

**Appendix Table S1: 23 unretracted randomised controlled trials at study start included in our previous systematic review**

| **Reference** | **Outcome 5 months after initial email** |
| --- | --- |
| J Neurol Neurosurg Psychiatry 1999;66:64-8 |  |
| J Neurol Neurosurg Psychiatry 2003;74:574-6 |  |
| J Neurol Neurosurg Psychiatry 2011;82:1390-3 |  |
| Aging Clin Exp Res 2005;17:157-63 |  |
| Bone 1998;23:291-6 |  |
| Bone 2002;31:114-8 |  |
| Bone 2005;36:61-8 |  |
| Bone 2006;39:1080-6 |  |
| J Neurol Sci 2002;202:65-8 |  |
| J Neurol Sci 2005;231:13-8 |  |
| J Musculoskelet Neuronal Interact 2012;12:136-43 |  |
| Cerebrovasc Dis 2005;20:187-92 |  |
| J Stroke Cerebrovasc Dis 2010;19:198-203 |  |
| J Stroke Cerebrovasc Dis 2011;20:41-6 |  |
| Kurume Med J 2011;57:117-24 | Retracted 13/4/17 |
| Am J Phys Med Rehabil 1999;78:457-63 |  |
| Am J Phys Med Rehabil 2004;83:298-303 |  |
| Osteoporos Int 2009;20:1233-40 |  |
| Osteoporos Int 2011;22:2735-42 |  |
| Stroke 1997;28:736-9 |  |
| Yonsei Med J 2005;46:750-8 |  |
| Yonsei Med J 2008;49:119-28 |  |
| Yonsei Med J 2009;50:474-81 |  |
